# Supplementary material for: The Segment Matters: Probable Reassortment of Tilapia Lake Virus (TiLV) Complicates Phylogenetic Analysis and Inference of Geographical Origin of New Isolate from Bangladesh
Source: Viruses. 2020 Feb 27;12(3):258. doi: 10.3390/v12030258 (PMC7150994; doi:10.3390/v12030258)

Qualimap Analysis Results

BAM QC analysis

Generated by Qualimap v.2.2.2-dev

2020/02/05 23:19:37

1. Input data & parameters

1.1. QualiMap command line

qualimap bamqc -bam TiLV_BD-2017.sorted.bam -nw 400 -hm 3

1.2. Alignment

Command line:	bwa mem -M -t 12 TiLV_BD-2017.fasta blacklist_paired_unaligned_liver_2.fq.1.gz blacklist_paired_unaligned_liver_2.fq.2.gz
Draw chromosome limits:	no
Analyze overlapping paired-end reads:	no
Program:	bwa (0.7.17-r1188)
Analysis date:	Wed Feb 05 23:19:35 UTC 2020
Size of a homopolymer:	3
Skip duplicate alignments:	no
Number of windows:	400
BAM file:	TiLV_BD-2017.sorted.bam

2. Summary

2.1. Globals

Reference size	10,123
Number of reads	14,054,812
Mapped reads	8,361 / 0.06%
Unmapped reads	14,046,451 / 99.94%
Mapped paired reads	8,361 / 0.06%
Mapped reads, first in pair	4,198 / 0.03%
Mapped reads, second in pair	4,163 / 0.03%
Mapped reads, both in pair	8,258 / 0.06%
Mapped reads, singletons	103 / 0%
Secondary alignments	30
Read min/max/mean length	30 / 300 / 196.26
Duplicated reads (estimated)	5,840 / 0.04%
Duplication rate	76.08%
Clipped reads	1,068 / 0.01%

2.2. ACGT Content

Number/percentage of A's	397,569 / 26.26%
Number/percentage of C's	358,046 / 23.65%
Number/percentage of T's	396,963 / 26.22%
Number/percentage of G's	361,583 / 23.88%
Number/percentage of N's	0 / 0%
GC Percentage	47.53%

2.3. Coverage

Mean	149.6117
Standard Deviation	96.1719

2.4. Mapping Quality

Mean Mapping Quality	59.98

2.5. Insert size

Mean	192.66
Standard Deviation	84.33
P25/Median/P75	138 / 172 / 227

2.6. Mismatches and indels

General error rate	0.4%
Mismatches	5,990
Insertions	33
Mapped reads with at least one insertion	0.39%
Deletions	92
Mapped reads with at least one deletion	1.1%
Homopolymer indels	43.2%

2.7. Chromosome stats

**Name**	**Length**	**Mapped bases**	**Mean coverage**	**Standard deviation**

BDS1	1620	146414	90.379	29.4942
BDS2	1448	132422	91.4517	24.1978
BDS3	1353	275476	203.6038	45.7528
BDS4	1226	56669	46.2227	17.8393
BDS5	1083	214726	198.2696	61.3932
BDS6	1024	181396	177.1445	50.1711
BDS7	758	69762	92.0343	31.2326
BDS8	637	183949	288.7739	71.5727
BDS9	531	181024	340.9115	145.8946
BDS10	443	72681	164.0655	44.3468

3. Results : Coverage across reference

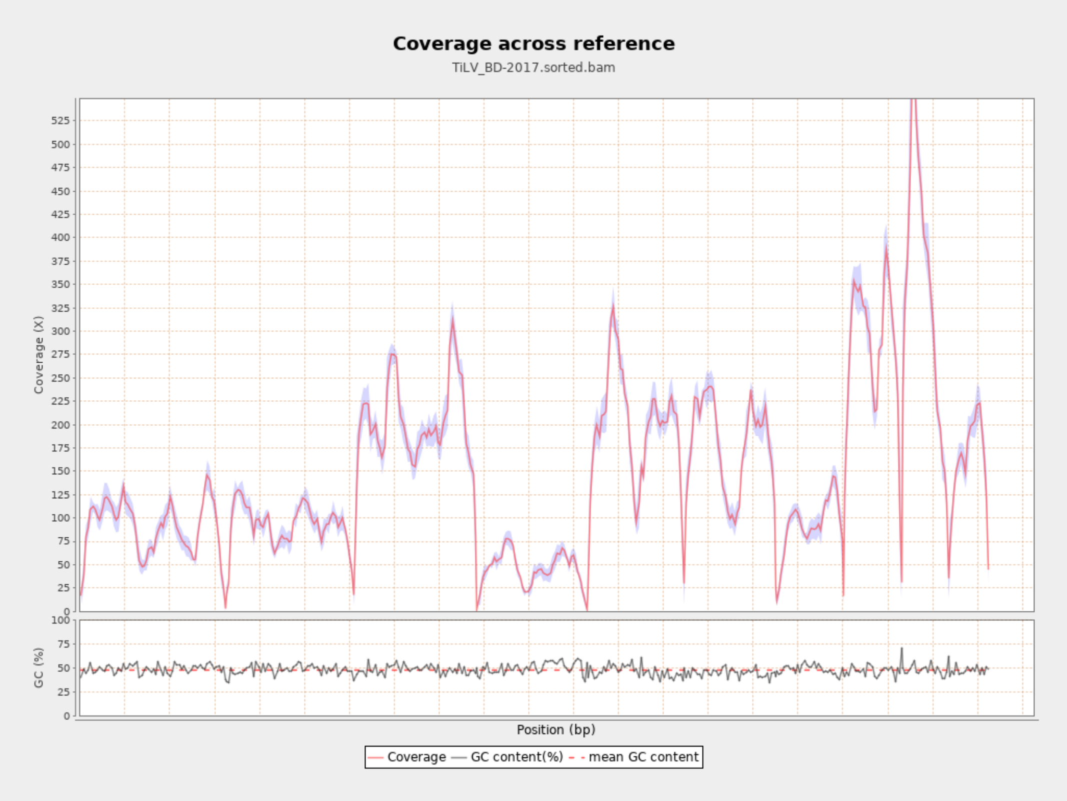

4. Results : Coverage Histogram

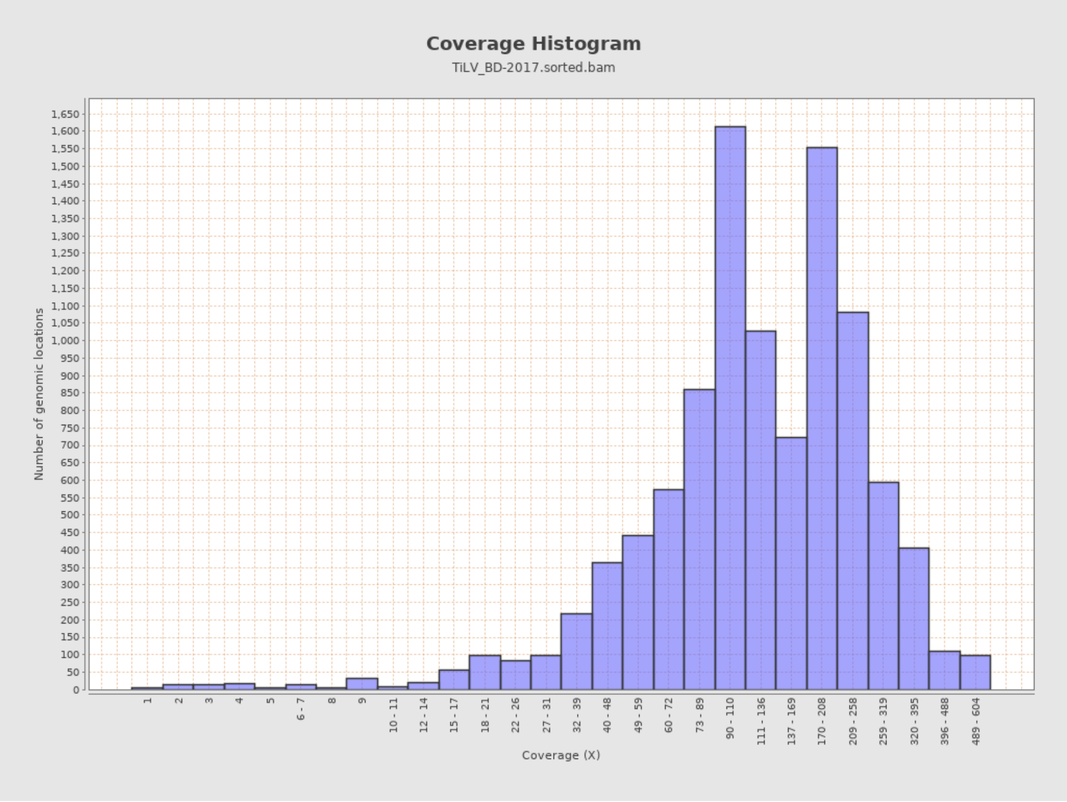

5. Results : Coverage Histogram (0-50X)

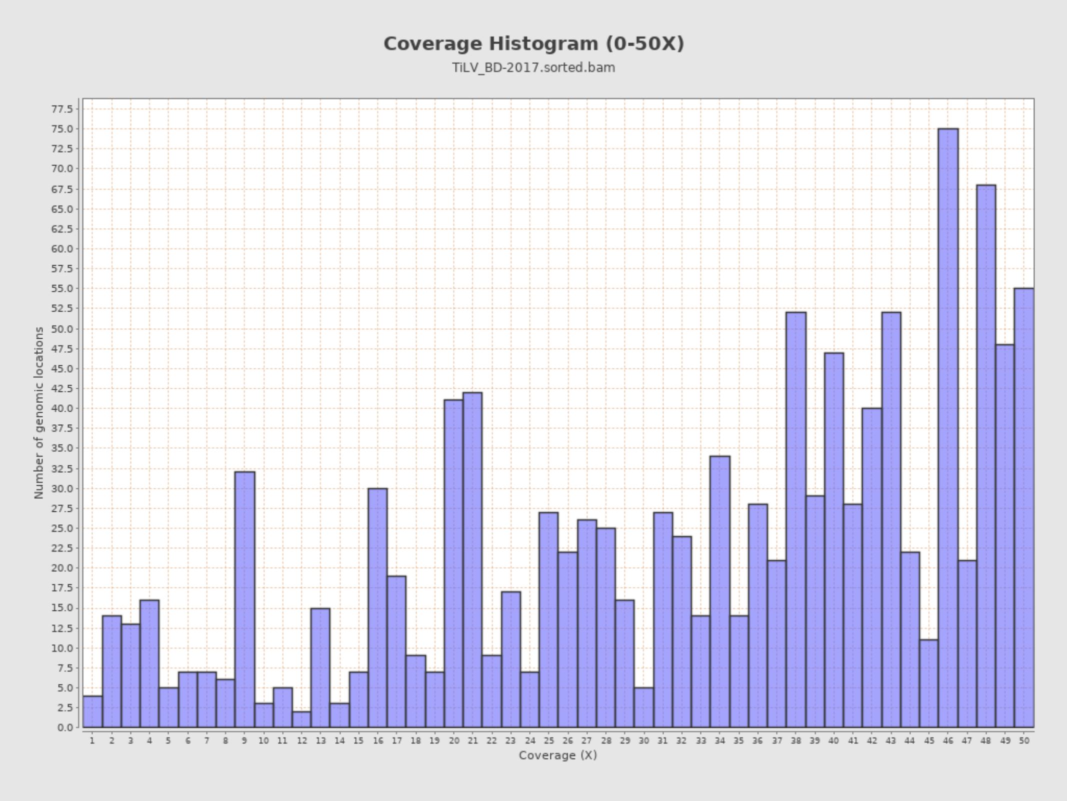

6. Results : Genome Fraction Coverage

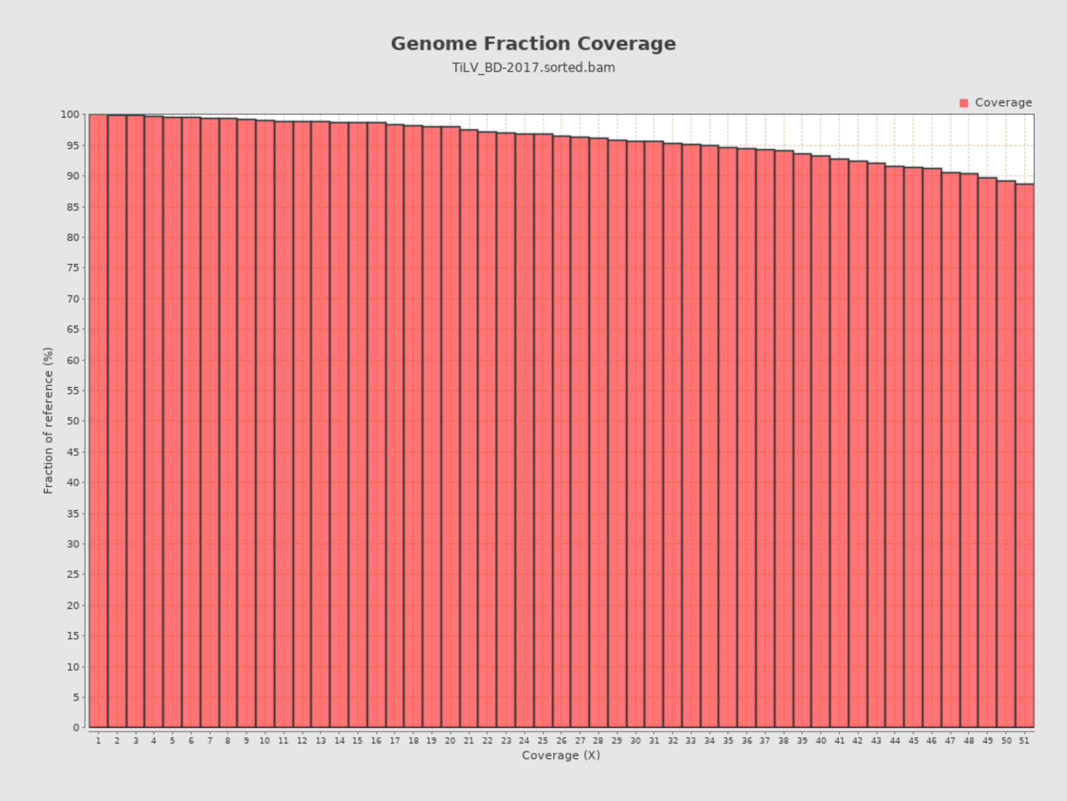

7. Results : Duplication Rate Histogram

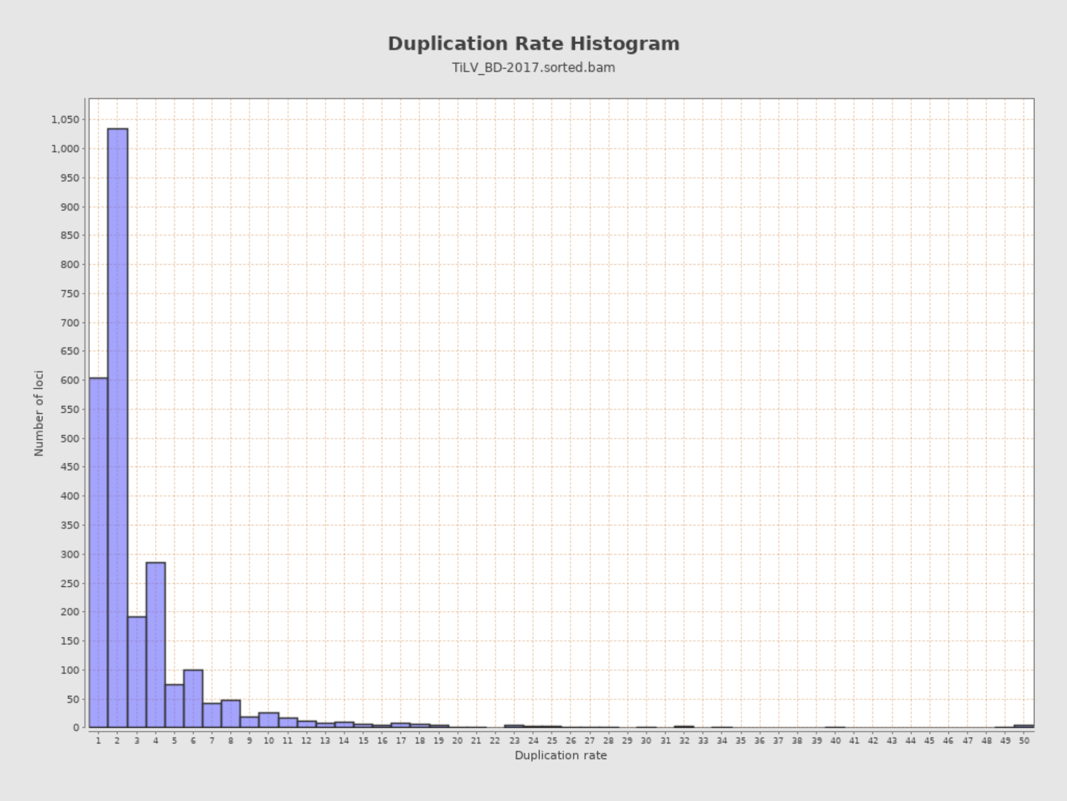

8. Results : Mapped Reads Nucleotide Content

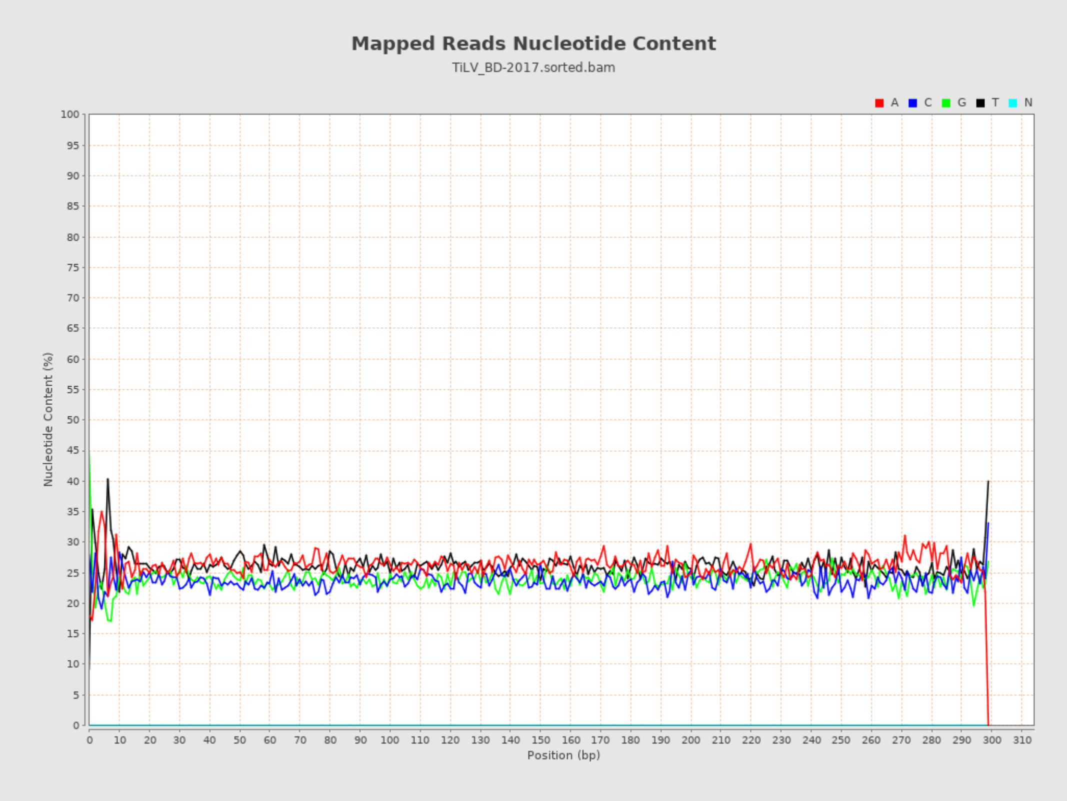

9. Results : Mapped Reads GC-content Distribution

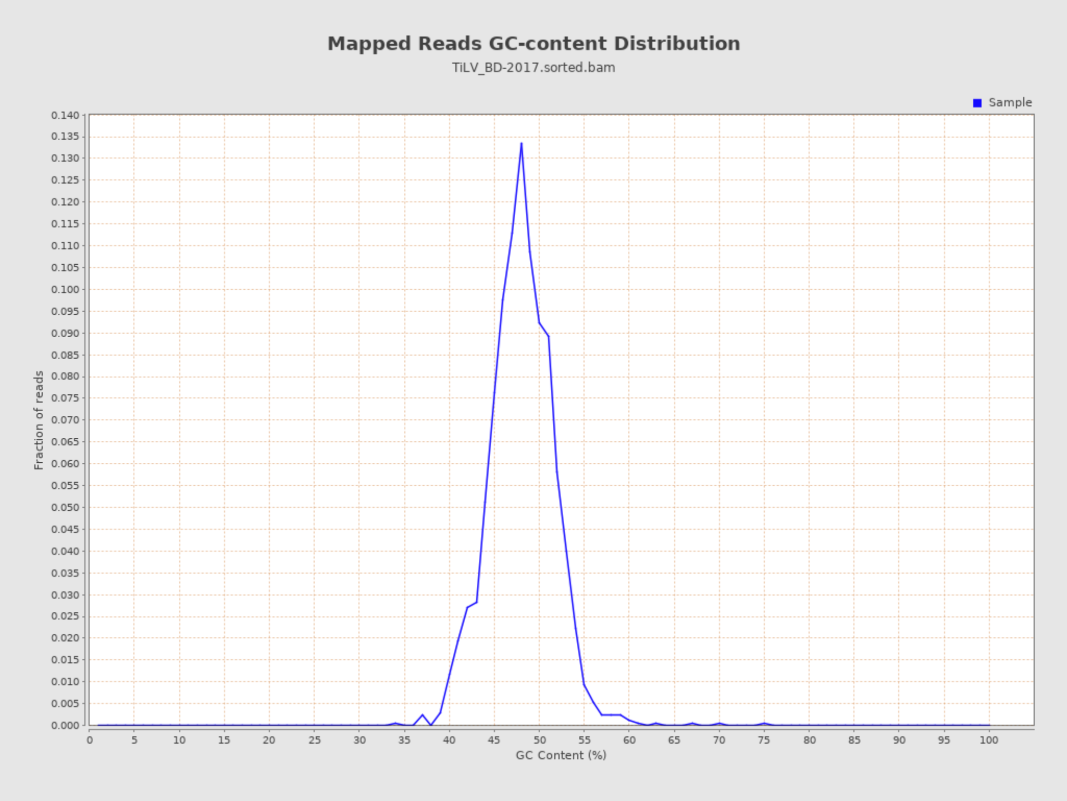

10. Results : Mapped Reads Clipping Profile

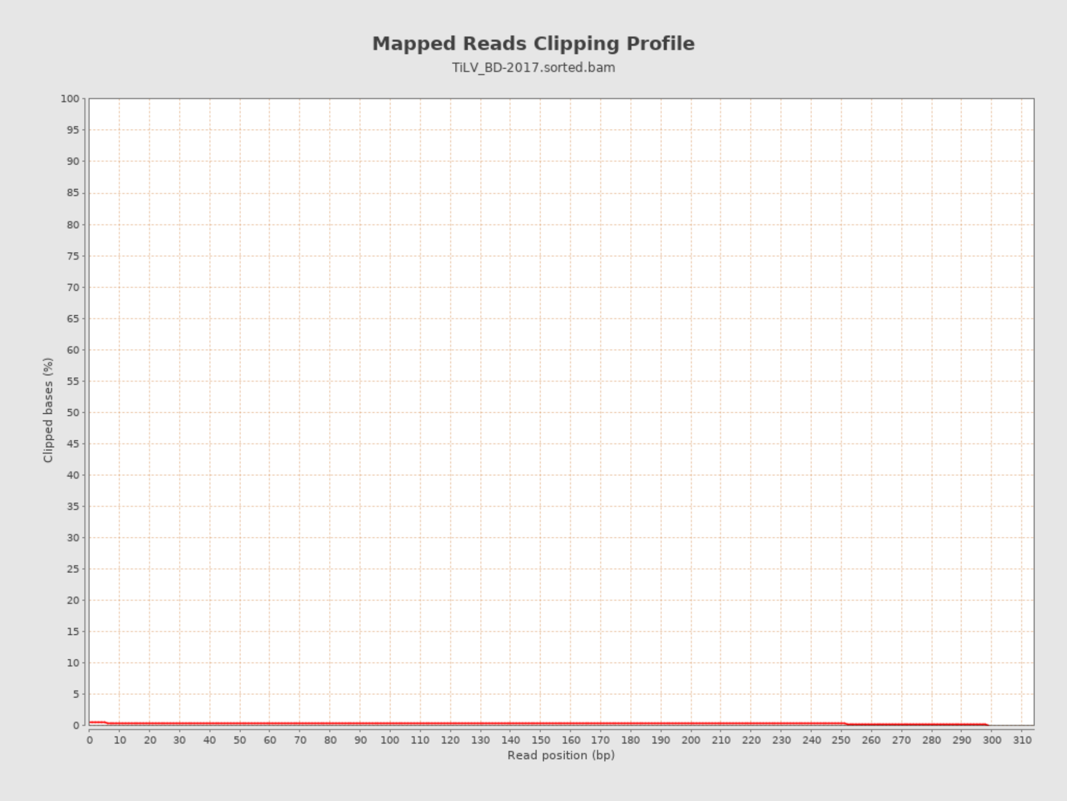

11. Results : Homopolymer Indels

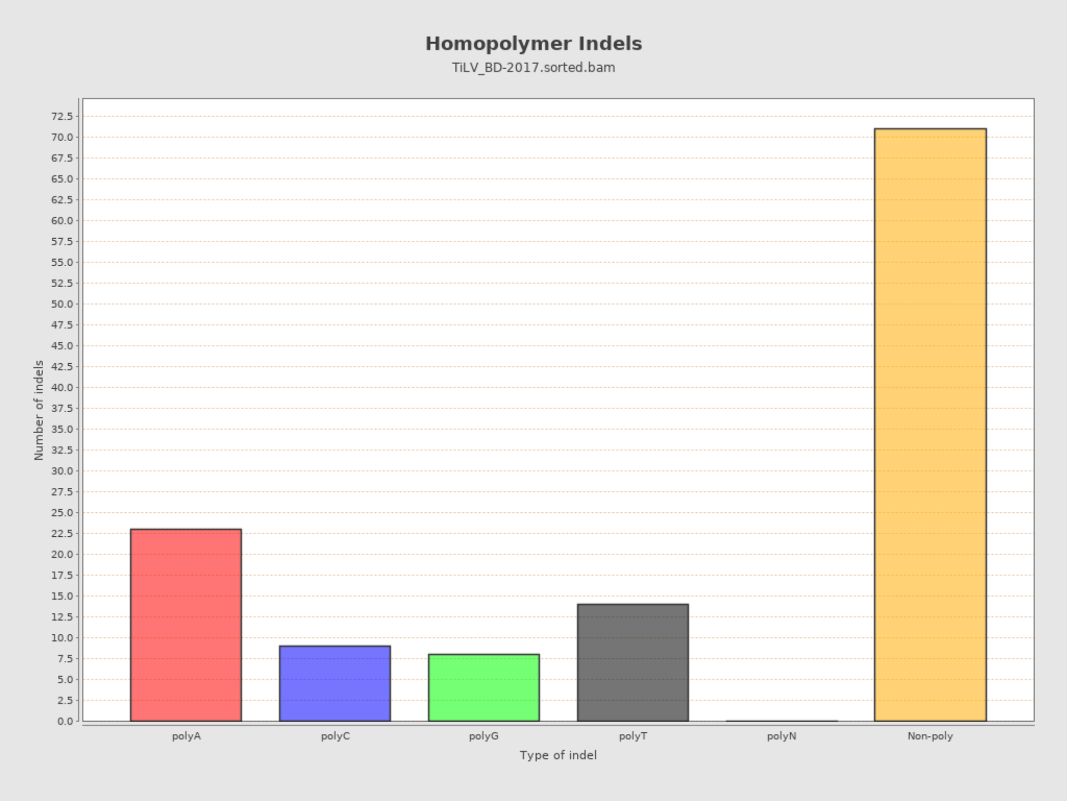

12. Results : Mapping Quality Across Reference

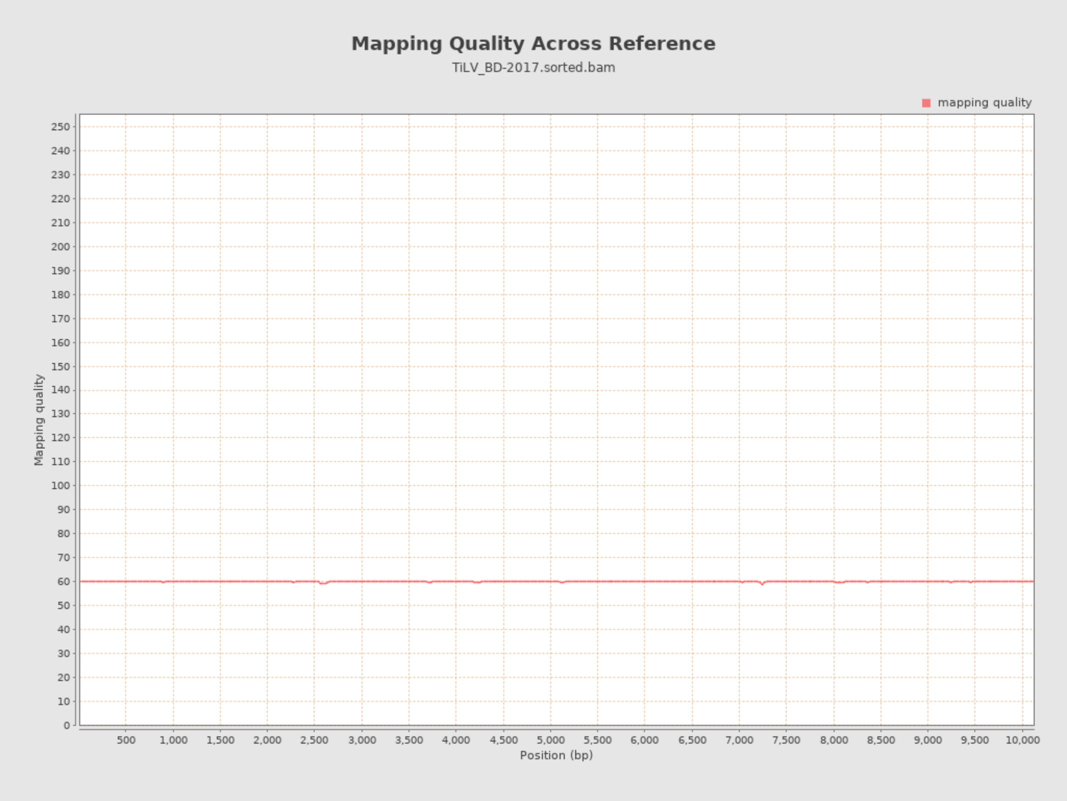

13. Results : Mapping Quality Histogram

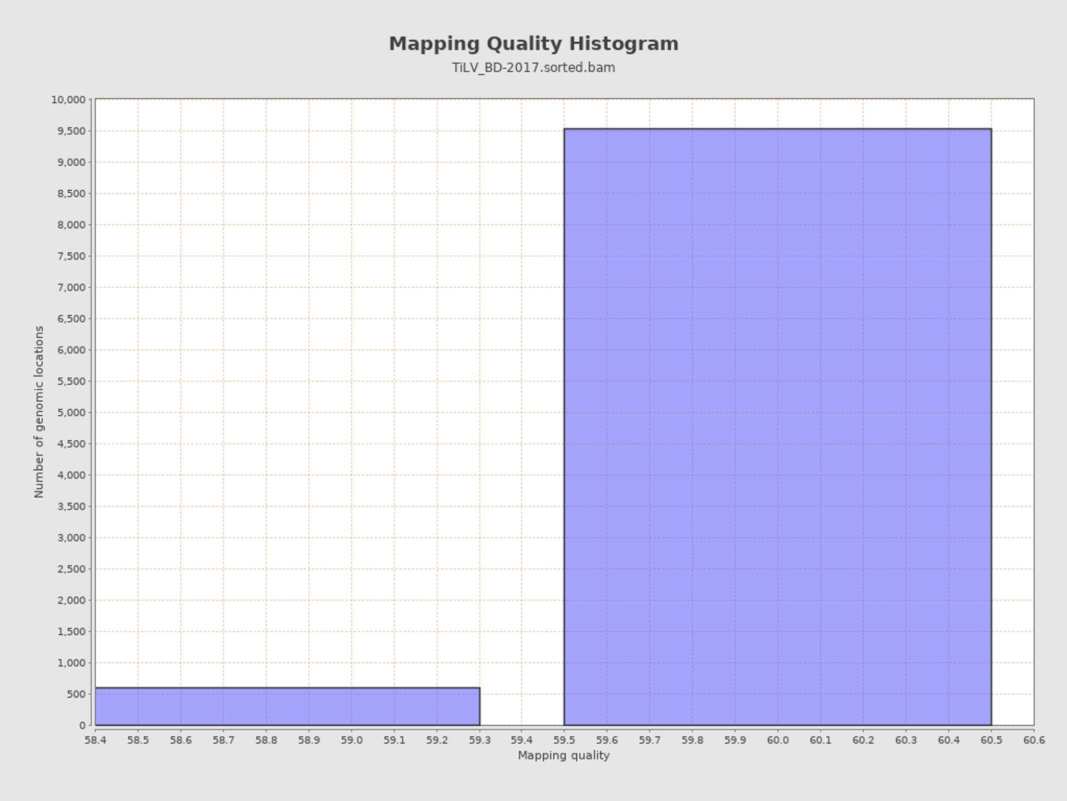

14. Results : Insert Size Across Reference

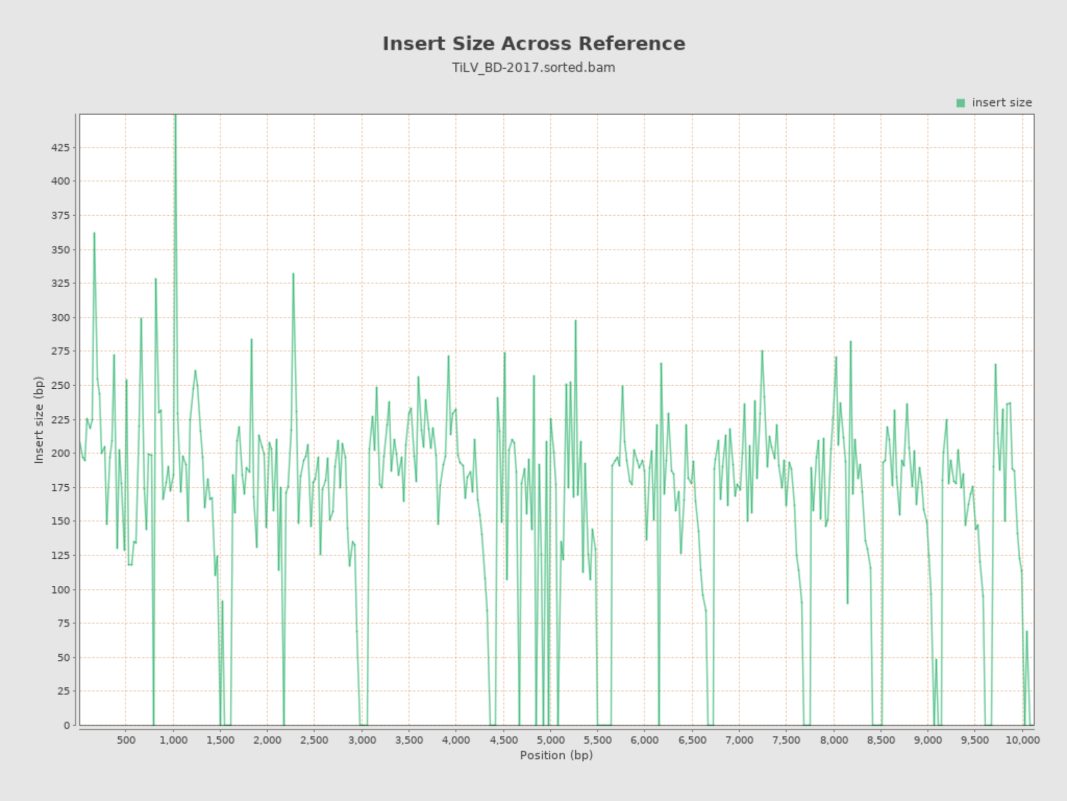

15. Results : Insert Size Histogram

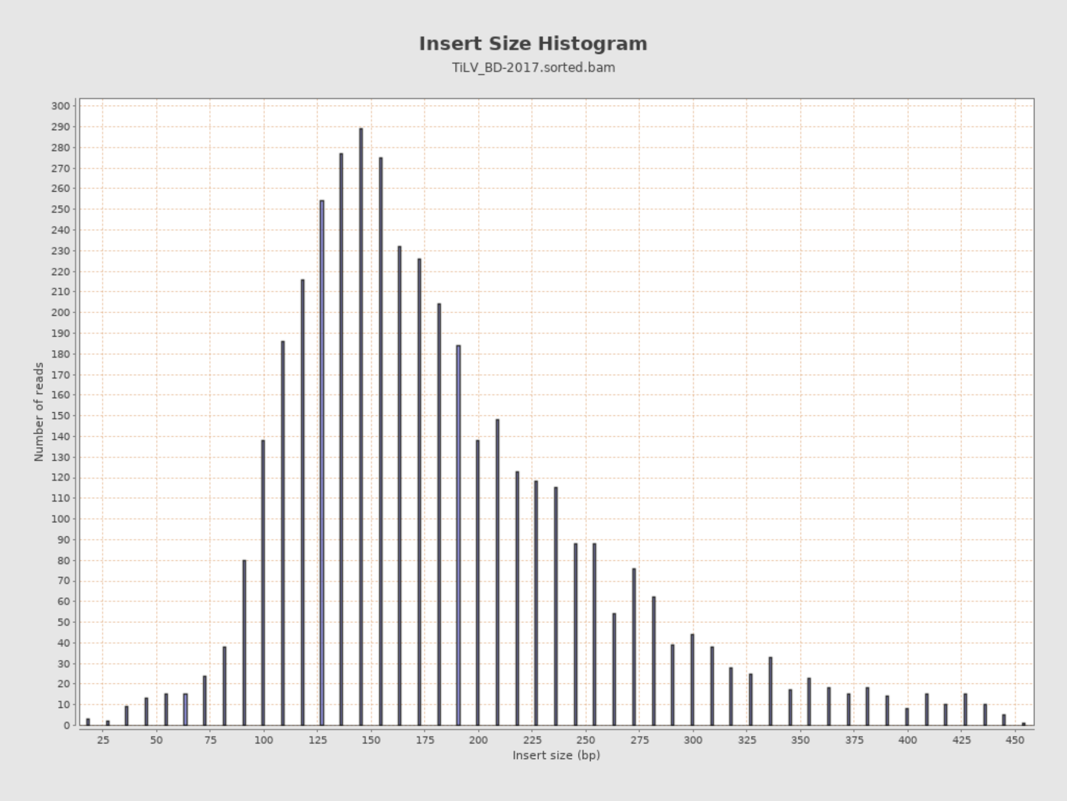

Supplement: Supplementary file 1 [file viruses-12-00258-s001.zip › TiLV_Suppl_data1_qualimap_TiLV_BD-2017.pdf]
