# Supplementary material for: The Segment Matters: Probable Reassortment of Tilapia Lake Virus (TiLV) Complicates Phylogenetic Analysis and Inference of Geographical Origin of New Isolate from Bangladesh
Source: Viruses. 2020 Feb 27;12(3):258. doi: 10.3390/v12030258 (PMC7150994; doi:10.3390/v12030258)
Supplement: Supplementary file 1 [file viruses-12-00258-s001.zip › TiLV_Suppl_info_BD-2017.pdf]

The segment matters: Probable reassortment of tilapia lake virus (TiLV) complicates phylogenetic analysis and inference of geographical origin of new isolate from Bangladesh

Dominique L. Chaput [1,*], David Bass [2,3], Md. Mehedi Alam [4], Neaz Al Hasan [4], Grant D. Stentiford [2,3], Ronny van Aerle [2,3], Karen Moore [5], John P. Bignell [3], Mohammad Mahfujul Haque [4,†] and Charles R. Tyler [1,2,*,†]

[1]Biosciences, Geoffrey Pope Building, University of Exeter, Exeter, Devon EX4 4QD, UK; [2]Centre for Sustainable Aquaculture Futures, University of Exeter, Exeter, Devon EX4 4QD, UK; [3]International Centre of Excellence for Aquatic Animal Health, Centre for Environment, Fisheries and Aquaculture Science (Cefas), Weymouth Laboratory, Weymouth, Dorset DT4 8UB, UK; [4]Department of Aquaculture, Bangladesh Agricultural University, Mymensingh-2202, Bangladesh; [5]Exeter Sequencing Service, Geoffrey Pope Building, University of Exeter, Exeter, Devon EX4 4QD, UK

*Correspondence: d.chaput@exeter.ac.uk (D.L.C.); c.r.tyler@exeter.ac.uk (C.R.T)

†These authors contributed equally to this work.

SUPPLEMENTAL INFORMATION:

Supplemental Methods

Supplemental Results

Supplemental Tables S1 to S4

Supplemental Figures S1 to S9

Supplemental References

Supplemental Methods

RNA extraction

Lysis and homogenisation were carried out on a TissueLyser II instrument (Qiagen, Venlo, Netherlands) in 2 mL tubes with a single 5 mm diameter steel bead for 2 x 2 min at 30 Hz. For skin/muscle samples, a proteinase K step was necessary for successful RNA extraction. Briefly, following lysis in 600 µL buffer, 400 µL lysate was mixed with 787 µL RNase-free water and 13 µL proteinase K solution (>600 mAU/mL, Qiagen, Venlo, Netherlands), then incubated at 55°C for 10 minutes. Following centrifugation at 10000 x g for 3 minutes, supernatant was moved to a clean tube, mixed with 0.5 vols 100% molecular grade EtOH (Thermo Fisher Scientific, Inc., Waltham MA, USA), and loaded onto an RNeasy spin column, with the subsequent washing and elution steps as described in the RNeasy Mini protocol.

Detection of TiLV by semi-nested RT-PCR assay

RNA (1 µg) was treated with RQ1 RNase-Free DNase (Promega) in a total volume of 10 µL, following the manufacturer's protocol. DNase was inactivated with 1 µL of RQ1 Stop Solution and a 10 minute incubation at 65°C. Reverse transcription of TiLV RNA was carried out with M-MLV reverse transcriptase (Promega, Madison WI, USA) and primers Nested ext-1 and ME1 (Supplemental Table S3), which are specific to a region of TiLV genomic segment 3. Primers (1 µL of 10 µM stocks) were added to the 1 µg RNA from the DNase step, for a total volume of 13 µL, and secondary structures were melted by heating to 70°C for 5

minutes followed by chilling on ice for 2 minutes. The following reagents were then added to the RNA-primer mixture: 5 µL M-MLV reaction buffer, 2 µL dNTP mix (10 mM each nucleotide), 4 µL water and 1 µL M-MLV reverse transcriptase (200U/µL), for a total volume of 25 µL. Reverse transcription was carried out at 50°C for 60 minutes.

The first PCR consisted of 1X GoTaq Flexi buffer (Promega, Madison WI, USA), 1.5 mM $MgCl_2$, 0.2 mM each dNTP, 0.4 µM each primer (Nested ext-1 and ME1), 1 unit GoTaq G2 Flexi DNA polymerase and 5 µL cDNA from the RT reaction (equivalent to 200 ng input RNA), in a total volume of 25 µL. The second PCR had the same concentrations of buffer, $MgCl_2$, primers ME1 and 7450/150R/ME2 (Table S3), dNTPs and polymerase, with 2.5 µL of the first PCR as template in a total volume of 50 µL. Thermocycling conditions for both PCRs were as follows: An initial denaturation at 94°C for 2 minutes, 25 cycles of 94°C for 30s, 60°C for 30s, and 72°C for 30s, then a final extension at 72°C for 5 minutes.

Sequencing of TiLV amplicons

A subset of amplicons from the RT-PCR assay (TiLV segment 3) was selected for sequencing. Fragments from the remaining nine segments were amplified as follows: RNA was treated with DNase and reverse transcribed to cDNA as described above, but with random hexamers instead of target-specific primers. PCRs were carried out with primers pairs for each segment (Table S4), the PCR recipe listed above, and the following thermocycling conditions: an initial

denaturation at 94°C for 2 minutes, 35 cycles of 94°C for 30s, 50°C for 30s, and 72°C for 60s, then a final extension at 72°C for 5 minutes. All amplicons were gel-purified with the Qiagen MinElute kit (Qiagen, Venlo, Netherlands) and submitted to Eurofins Genomics (Ebersberg, Germany) for Sanger sequencing. Chromatograms were viewed and trimmed in 4Peaks (Nucleobytes, Amsterdam, The Netherlands), forward and reverse reads were assembled in AliView v1.18 [1], and the consensus sequences were searched against GenBank using BLAST.

Histopathology

Four Nile tilapia were sampled for histopathology (three diseased, one asymptomatic). Following euthanasia by Schedule 1, fish were observed for clinical signs of disease, visceral organs were removed (liver, heart, kidney, spleen, gonad, intestine) and gill, skin and muscle also sampled for formalin fixed paraffin embedded (FFPE) histology. Samples of tissues were placed into 10% Neutral Buffered Formalin (NBF) prior to histological processing in a vacuum infiltration processor using standard histological protocols and subsequently embedded in paraffin wax. Sections of 3-4 µm were obtained using a rotary microtome and stained with haematoxylin and eosin (HE). Slides were examined for signs of infectious disease and general health using a Nikon Eclipse Ni-U microscope (Nikon Corporation, Tokyo, Japan). Slides were digitally scanned using a Carl Zeiss Axio Scan Z1 digital slide scanner (Zeiss Group, Oberkochen, Germany) and uploaded to the online Cefas Digital Pathology Portal.

Supplemental Results

Histopathology

Following histological examination of tissues, two fish (F1 and F2) exhibited significant pathological changes consistent with previous descriptions of Tilapia lake virus (TiLV) disease (Ferguson *et al.,* 2014). Inflammation was observed associated with extensive pancreatitis throughout the liver (Figure S4). Hepatocellular changes were varied and extensive, with a significant loss in normal tissue architecture. Many hepatocytes exhibited signs of progressive necrosis characterised by cytoplasmic degeneration. The cytoplasm possessed little cellular integrity with a foam-like appearance that was disassociated from the plasma membrane. Hepatocellular regeneration also appeared to be widespread (Figure S5). Melanomacrophage aggregates of varying size were seen throughout the liver and apoptosis could also be seen on occasion. Cytoplasmic inclusion bodies, appearing as eosinophilic proteinaceous material, possibly phagocytosed red blood cells, was observed in a significant number of hepatocytes throughout the liver (Figure S6). Brown lipofuscin-like pigment granules, presumably lysosomal in origin, were also observed in a significant number of hepatocytes throughout the liver. The formation of multinucleated syncytial giant cells following fusion of viral infected hepatocytes was particularly evident. Syncytia were dispersed throughout the liver appearing as large cells containing 10-20+ nuclei (Figure S7). The formation of smaller early stage syncytia could also be seen containing fewer nuclei e.g. 3-4. Early stage syncytia were relatively small (15-20 µm) and possessed a relatively condensed

cytoplasm. Late stage syncytia were large (60-70 μm) with an amorphous cytoplasm with indistinct plasma membrane. No necrotic changes were observed within the gastric glands of intestinal tract.

Supplemental Tables

Table S1. Physical/chemical parameters of affected pond water

Parameter	Mean ± SD (n=3)
Salinity (ppm)	287.3 ± 4.6
Temperature (°C)	32.6 ± 0.2
pH	7.66 ± 0.06
Dissolved oxygen (mgL^{-1})	12.4 ± 1.4

Table S2. Fish sampled from affected pond in Trishal Upazila, Bangladesh, July 6, 2017.

#	Species	Health status	Sex	Mass (g)	Length (mm)	Dissection notes
**F1**	Tilapia	Diseased	M	492	360	Fin rot, small liver, tiny stomach
**F2**	Tilapia	Diseased	M	450	255	Small liver, tiny stomach
**F3**	Pangasius	Asymptomatic	M	657	370	
**F4**	Common carp	Diseased	M	208	213	Swollen abdomen
**F5**	Rohu	Asymptomatic	F	55	170	
**F6**	Tilapia	Asymptomatic	M	456	262	
**F7**	Tilapia	Diseased	M	238	245	Small liver, tiny stomach

Table S3. Primers used in semi-nested RT-PCR assay for TiLV, from Dong et al. (2017), modified from Kembou Tsofack et al. (2017) and Eyngor et al. (2014).

Primer name	Sequence (5'->3')	
ME1	GTTGGGCACAAGGCATCCTA	Reverse transcription, first and second PCR
Nested ext-1	TATGCAGTACTTTCCCTGCC	Reverse transcription, first PCR
7450/150R/ME2	TATCACGTGCGTACTCGTTCAGT	Second PCR only

Table S4. Primers used to amplify a fragment of each of the ten TiLV segments.

Seg.	Primer names	Primer sequence (5'->3')	Product length (nt)	Ref.
1	TiLV-Seg1-F<br>TiLV-Seg1-R	TCATTCGCCTATATAGTTAC<br>TTAATTACGCACTATTACTG	1614	Dong et al. 2017 [2]
2	NORTH-S2-F1<br>NORTH-S2-R1	CGGAAATTCTCAACCCTCATA<br>AACAAGCGCTTTCAGTGAGGAAC	489	Bacharach et al. 2016 [5]
3	Nested ext-1<br>ME1	TATGCAGTACTTTCCCTGCC<br>GTTGGGCACAAGGCATCCTA	415	Eyngor et al. 2014 [4] & Kembou Tsofack et al. 2017 [3]
4	NORTH-S4-F1<br>NORTH-S4-R1	CTGTTTCTGAATGGCAATGCACT<br>ATGCAGCTATGATTCAGATGATG	556	Bacharach et al. 2016 [5]
5	TiLV-Seg5-F<br>TiLV-Seg5-R	TTTTTCTCAGTTTACCACTC<br>TTATCTCAGACTCCAATAGC	1073	Dong et al. 2017 [2]
6	NORTH-S6-F1<br>NORTH-S6-R1	CCACACGATAGGACCTATAGTGT<br>GGACTTGATGGTAGCAACGAAG	744	Bacharach et al. 2016 [5]
7	NORTH-S7-F1<br>NORTH-S7-R1	CTACCATCCTTAGTGAACGGTACT<br>TCCCCAAGGACAGCGGAAGTCA	659	Bacharach et al. 2016 [5]
8	NORTH-S8-F1<br>NORTH-S8-R1	GAGTAGCTTACCTCCCTGGGGAA<br>CAAGGGAAGCTCTACGATTTCA	539	Bacharach et al. 2016 [5]
9	TiLV-Seg9-F<br>TiLV-Seg9-R	ACGTCCTTAAAGTCATACTT<br>ACAAGTCCGATTACTTTTTC	522	Dong et al. 2017 [2]
10	NORTH-S10-F1<br>NORTH-S10-R1	TTCCCTCTGACACCCTGTATAGT<br>TCAAGTGACAGTGACTCGGG	391	Bacharach et al. 2016 [5]

Supplemental Figures

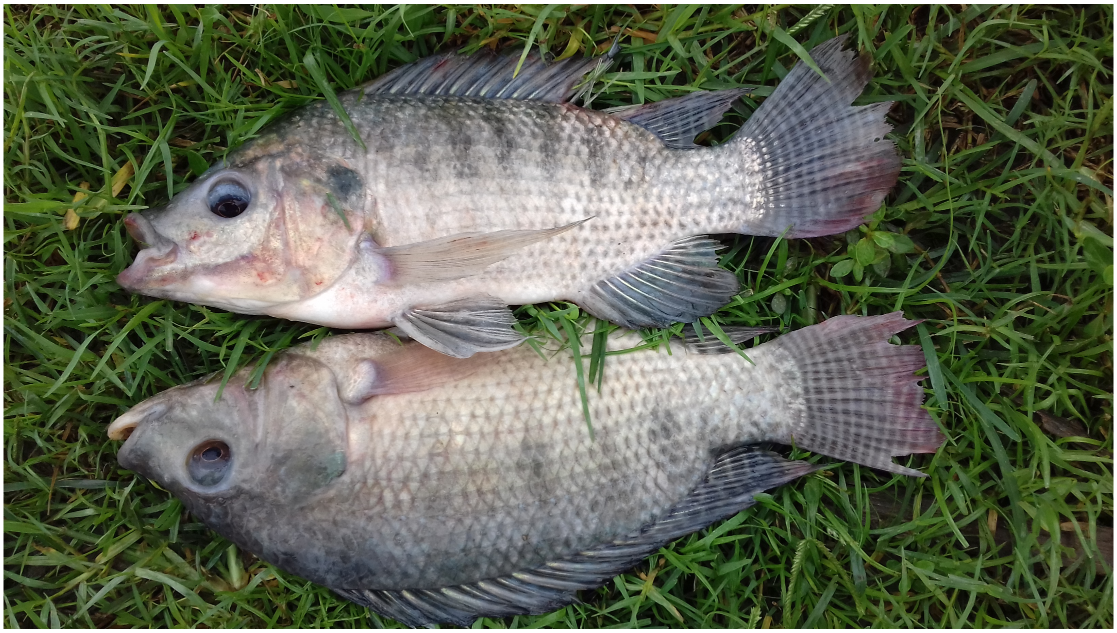

Figure S1. Diseased tilapia from affected pond in Trishal Upazila, Bangladesh (photo credit: Dr M. Mahfujul Haque).

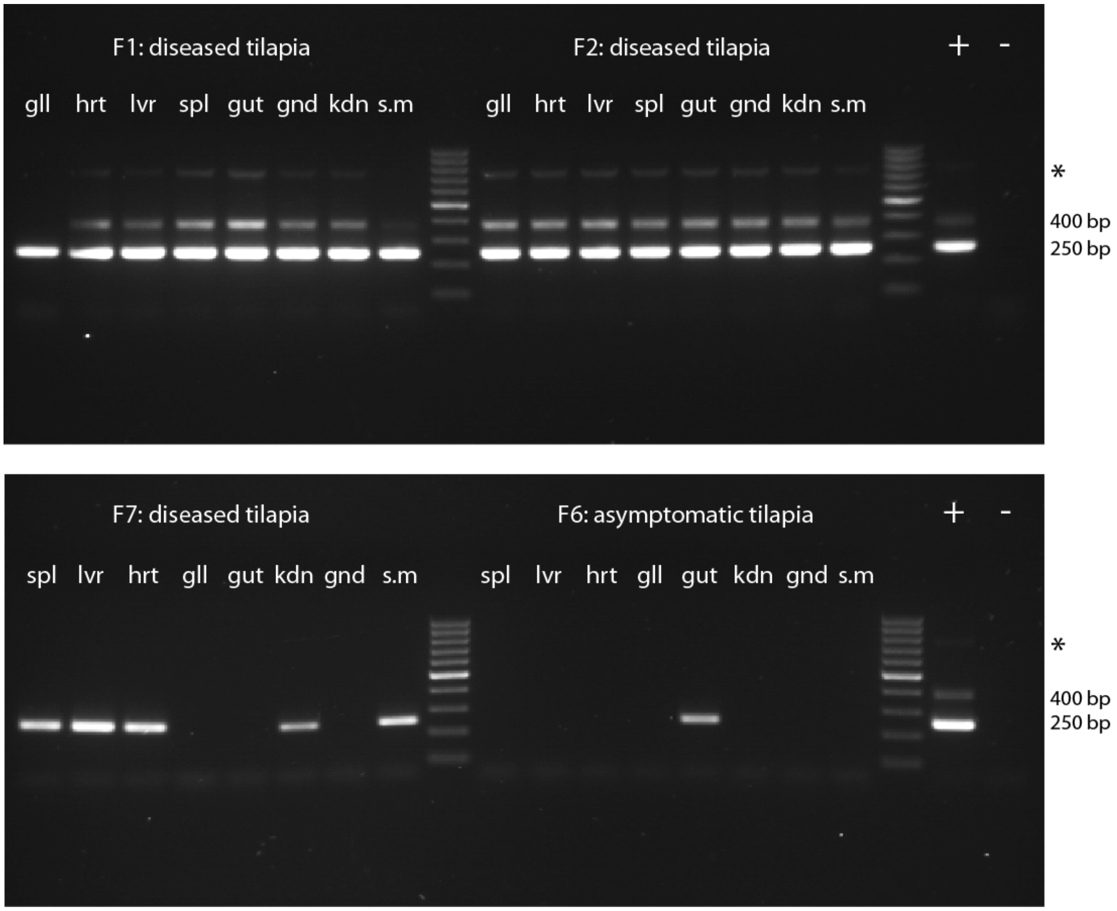

Figure S2. Detection of amplicons consistent with TiLV in tilapia tissues from affected pond in Trishal Upazila, Bangladesh, using the semi-nested RT-PCR approach of Dong *et al.* (2017). Light to moderate infection gives a single band of 250 bp. Severe infection gives multiple bands at 250 bp and ~400 bp. gll=gill, hrt=heart, lvr=liver, spl=spleen, gnd=gonad, kdn=kidney, s.m=skin+muscle. *Band of ~750 bp is likely from cross-hybridization of amplified products. Positive control consisted of PCR products from liver of F2 (diseased tilapia), previously confirmed as TiLV by Sanger sequencing. Size marker (GeneRuler 100 bp DNA Ladder, ThermoFisher) spans 100-1000 bp in 100 bp increments.

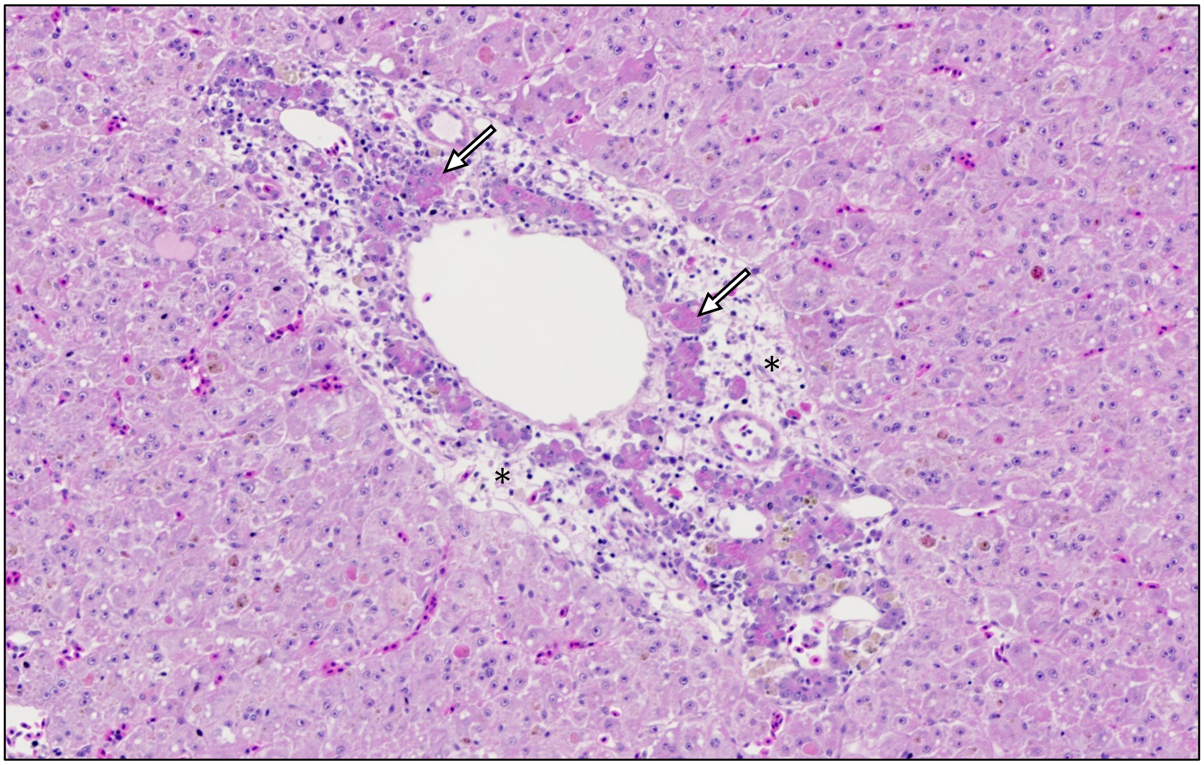

Figure S3. Necrosis of pancreatic acinar cells (*) and accompanying inflammation (pancreatitis). Note the presence of few remaining normal acinar cells (arrows).

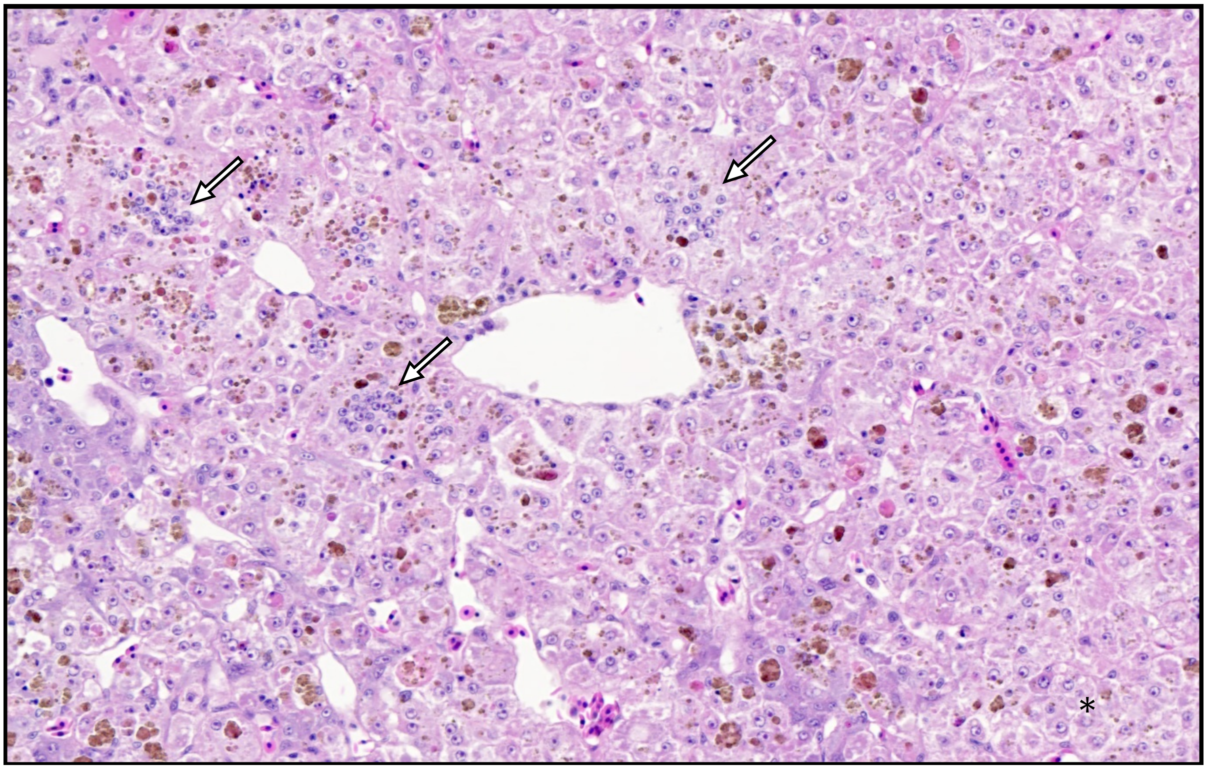

Figure S4. Extensive loss of normal liver tissue architecture was evident throughout the liver accompanied by a combination of hepatocellular necrosis (*) and formation of multinucleated syncytia (arrow). Widespread hepatocellular regeneration is also apparent.

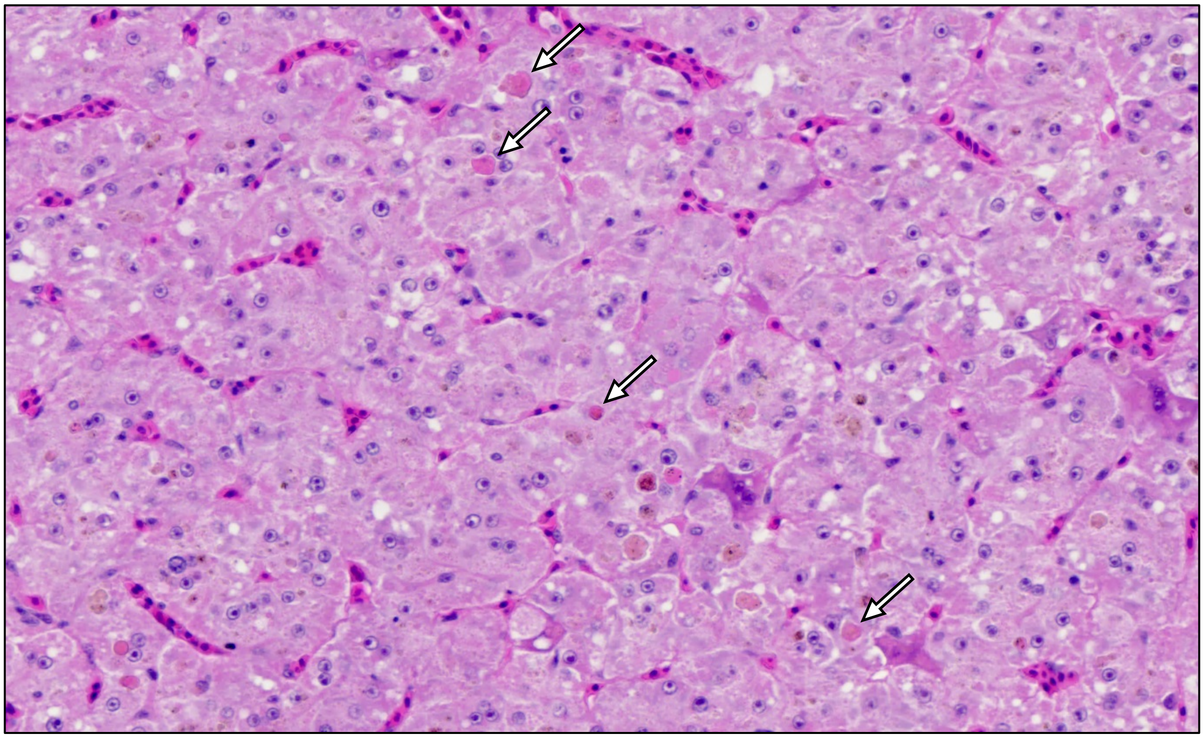

Figure S5. Eosinophilic cytoplasmic inclusion bodies within hepatocytes appeared to be proteinaceous and occurred in varying sizes extensively throughout the liver (arrow).

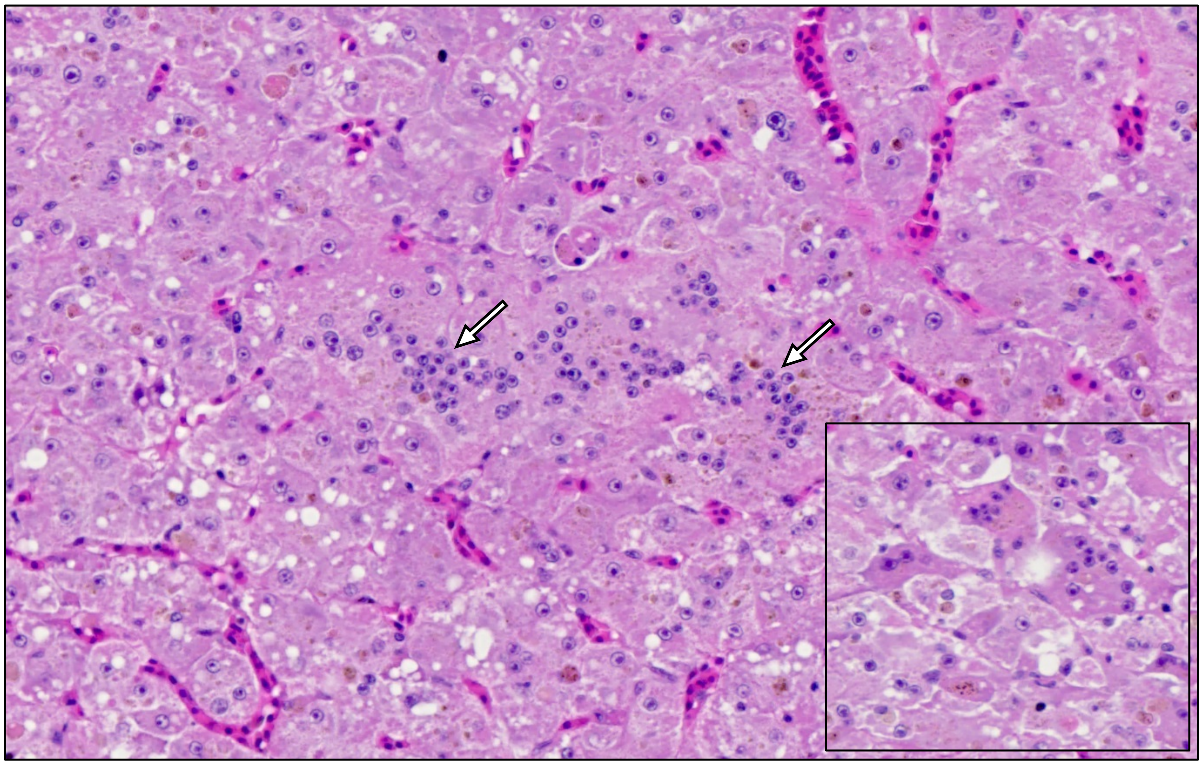

Figure S6. Large multinucleated syncytia (arrows) in liver tissue often contained a significant number of nuclei. Syncytia containing upwards of 15-20 nuclei were observed frequently. Early stage syncytia could also be seen with fewer nuclei present (inset).

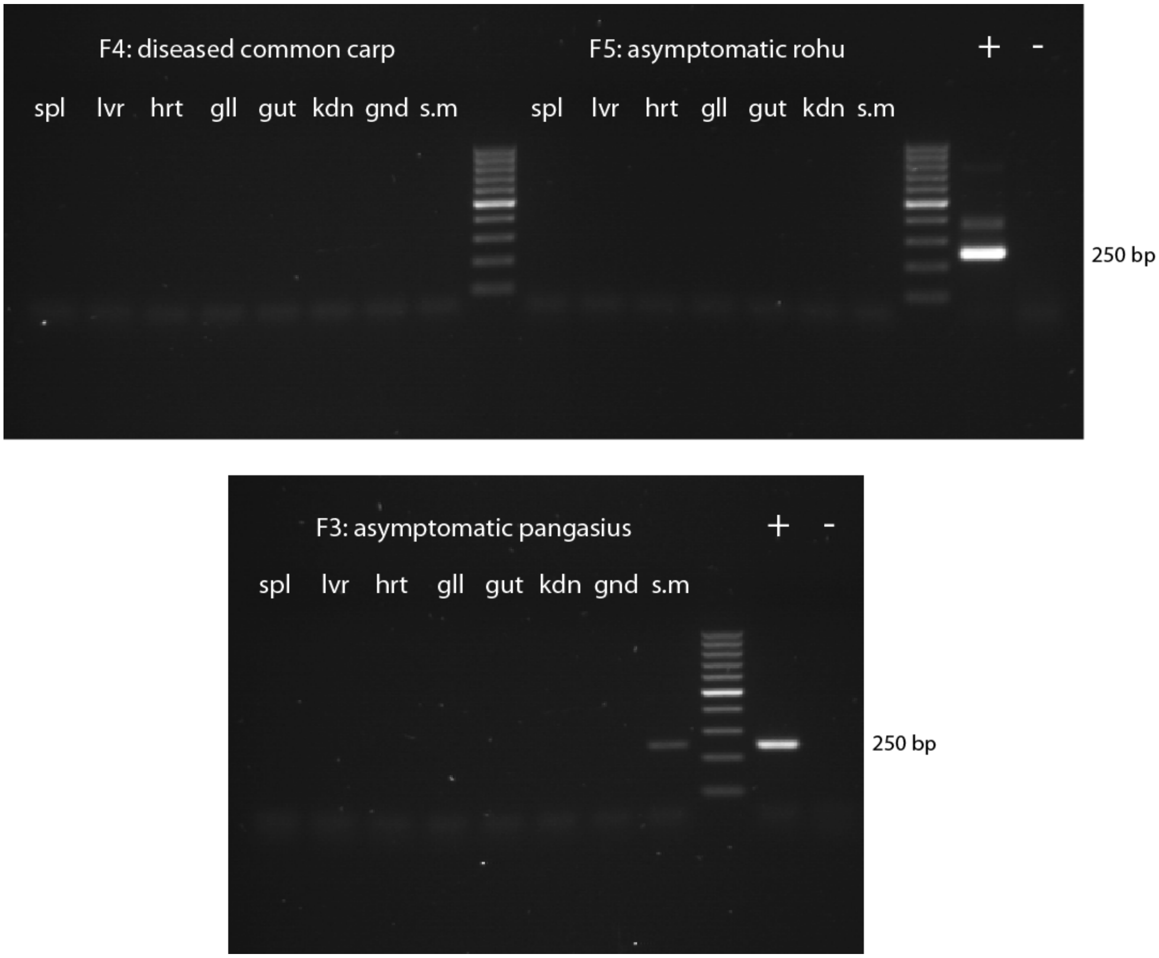

Figure S7. Absence of detectable TiLV amplicons in common carp, rohu and pangasius tissues from the affected pond in Trishal Upazila, Bangladesh, with the exception of a faint RT-PCR band in the pangasius skin/muscle sample. Light to moderate infection gives a single band of 250 bp. Positive controls consisted of PCR products from diseased tilapia livers of F2 (top gel) and F7 (bottom gel), previously confirmed as TiLV by Sanger sequencing. Size marker (GeneRuler 100 bp DNA Ladder, ThermoFisher) spans 100-1000 bp in 100 bp increments.

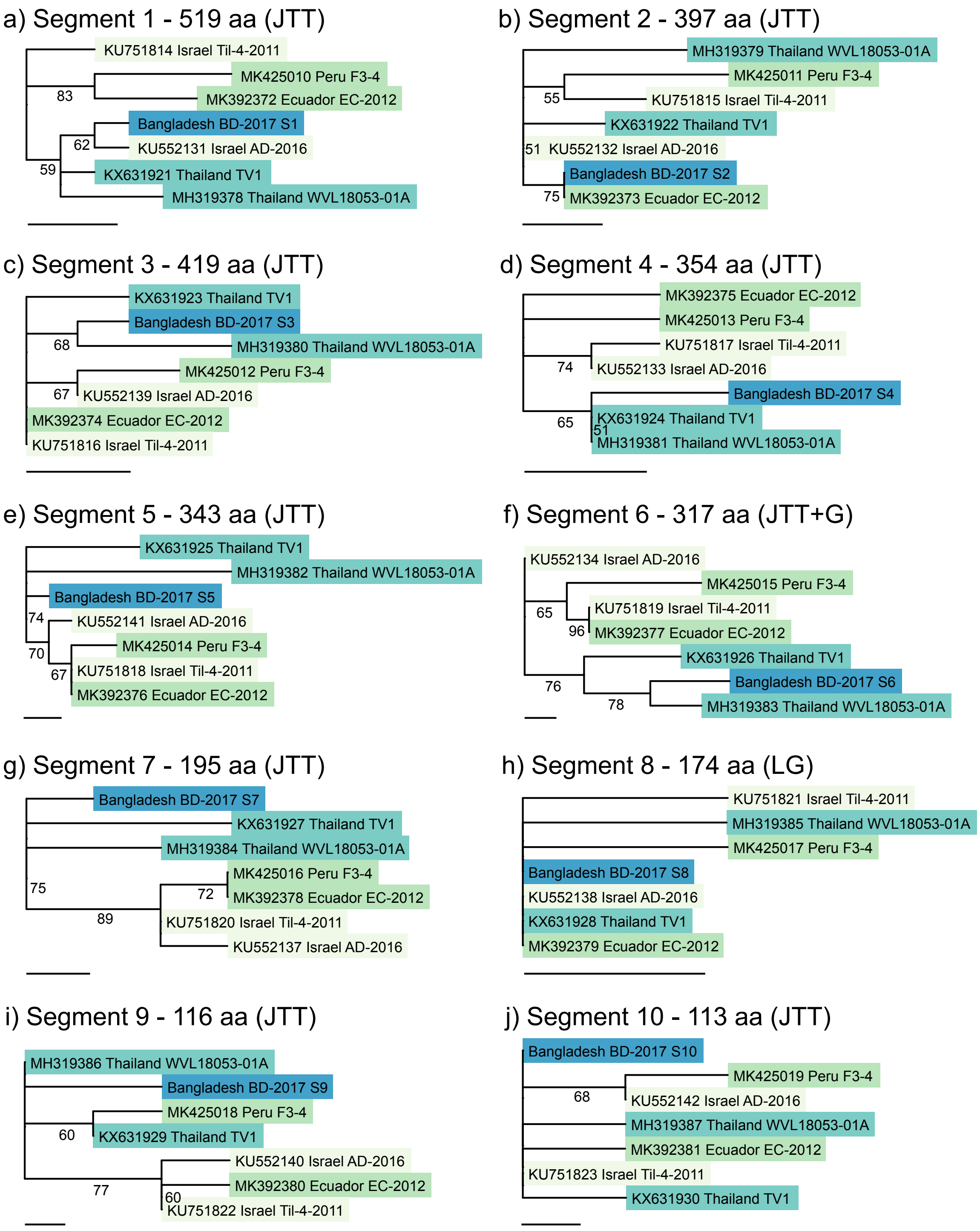

Figure S8. Maximum likelihood phylogenetic trees based on predicted amino acid sequence alignments of the ten TiLV segments, using only isolates for which all ten segments have been sequenced. Alignment lengths and best-fitting amino acid substitution models are shown in panel headings (JTT = Jones-Taylor-Thornton model, LG = Le and Gascuel 2008 model, +G = gamma-distributed rate heterogeneity). Bootstrap values were calculated from 500 replicates, and percent support is shown on nodes where values exceed 50%. Scale bars show 0.005 substitutions per site. Colours show isolates from the same region.

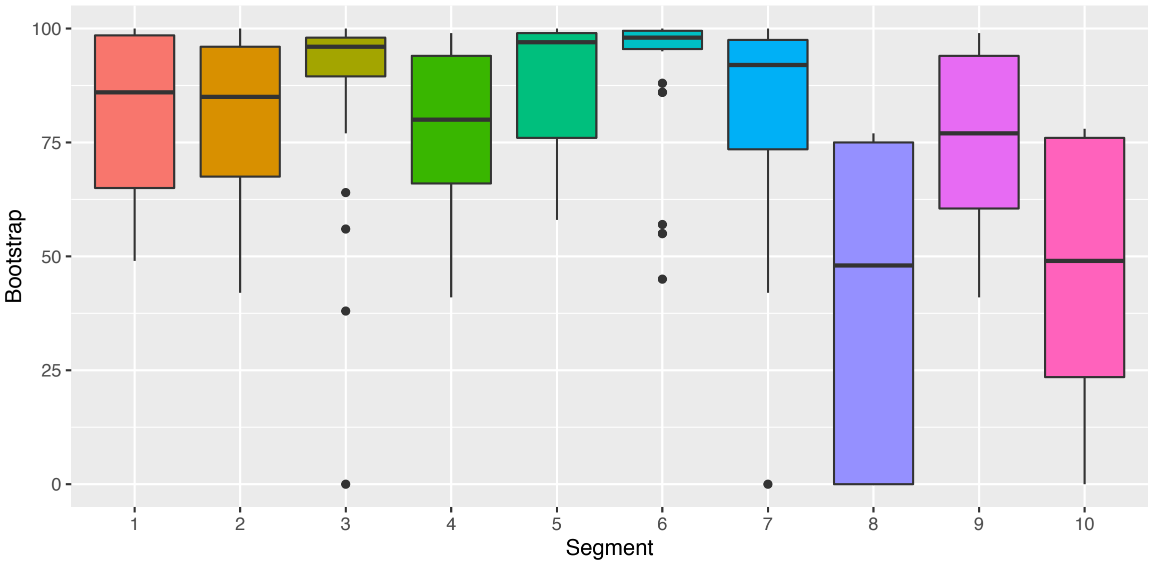

Figure S9. Bootstrap values of neighbour-joining trees constructed from segments 1-10 of 35 TiLV isolate quartets. Values were calculated from 1000 replicates.

References

1. Larsson, A. AliView: A fast and lightweight alignment viewer and editor for large datasets. *Bioinformatics* **2014**, *30*, 3276–3278.
2. Dong, H.T.; Siriroob, S.; Meemetta, W.; Santimanawong, W.; Gangnonngiw, W.; Pirarat, N.; Khunrae, P.; Rattanarojpong, T.; Vanichviriyakit, R.; Senapin, S. Emergence of tilapia lake virus in Thailand and an alternative semi-nested RT-PCR for detection. *Aquaculture* **2017**, *476*, 111–118.
3. Kembou Tsofack, J.E.; Zamostiano, R.; Watted, S.; Berkowitz, A.; Rosenbluth, E.; Mishra, N.; Briese, T.; Lipkin, W.I.; Kabuusu, R.M.; Ferguson, H.; et al. Detection of tilapia lake virus in clinical samples by culturing and nested reverse transcription-PCR. *Clin. Vet. Microbiol.* **2017**, *55*, 759–767.
4. Eyngor, M.; Zamostiano, R.; Tsofack, J.E.K.; Berkowitz, A.; Bercovier, H.; Tinman, S.; Lev, M.; Hurvitz, A.; Galeotti, M.; Bacharach, E.; et al. Identification of a novel RNA virus lethal to tilapia. *J. Clin. Microbiol.* **2014**, *52*, 4137–4146.
5. Bacharach, E.; Mishra, N.; Briese, T.; Zody, M.C.; Kembou Tsofack, J.E.; Zamostiano, R.; Berkowitz, A.; Ng, J.; Nitido, A.; Corvelo, A.; et al. Characterization of a Novel Orthomyxo-like Virus Causing Mass Die-Offs of Tilapia. *MBio* **2016**, *7*, 1–7.
